# Does reading a book in bed make a difference to sleep in comparison to not reading a book in bed? The People’s Trial*—*an online, pragmatic, randomised trial

**DOI:** 10.1186/s13063-021-05831-3

**Published:** 2021-12-04

**Authors:** Elaine Finucane, Ann O’Brien, Shaun Treweek, John Newell, Kishor Das, Sarah Chapman, Paul Wicks, Sandra Galvin, Patricia Healy, Linda Biesty, Katie Gillies, Anna Noel-Storr, Heidi Gardner, Mary Frances O’Reilly, Declan Devane

**Affiliations:** 1 School of Nursing and Midwifery, National University of Ireland Galway, Galway, Ireland; 2 Health Research Board-Trials Methodology Research Network, School of Nursing and Midwifery, National University of Ireland Galway, Galway, Ireland; 3 Evidence Synthesis Ireland, School of Nursing and Midwifery, National University of Ireland Galway, Galway, Ireland; 4 J.E. Cairnes School of Business & Economics, National University of Ireland Galway, Galway, Ireland; 5 Health Services Research Unit, Health Sciences Building, University of Aberdeen, Forester Hill, Aberdeen, AB25 2ZD UK; 6 School of Mathematics, Statistics and Applied Mathematics, National University of Ireland Galway, Galway, Ireland; 7 Cochrane UK, hosted by Oxford University Hospitals NHS Foundation Trust, and funded by the National Institute for Health Research, London, UK; 8 Wicks Digital Health, Lichfield, England, UK; 9 Radcliffe Department of Medicine, University of Oxford, Oxford, UK; 10 Formerly - Nursing and Midwifery Planning and Development Unit, West Mid-West, Merlin Park University Hospital, Galway, Ireland; 11 Cochrane Ireland, National University of Ireland Galway, Galway, Ireland

**Keywords:** Randomised trial, Public engagement, Online, Methodology, Research co-production, Sleep, We wrote this report using a plain language format. We did this in response to how people told us they wanted the results of The Reading Trial to be shared (phase vii of The People’s Trial).

## Abstract

**Background:**

The best way of comparing healthcare treatments is through a randomised trial. In a randomised trial, we compare something (a treatment or intervention) to something else, often another treatment. Who gets what is decided at random, meaning everyone has an equal chance of getting any of the treatments. This means any differences found can be put down to the treatment received rather than other things, such as where people live, or health conditions they might have.

*The People’s Trial* aimed to help the public better understand randomised trials by inviting them to design and carry out a trial. The question chosen by the public for *The People’s Trial* was:

‘Does reading a book in bed make a difference to sleep, in comparison to not reading a book in bed?’

This paper describes that trial, called ‘The Reading Trial’.

**Methods:**

The Reading Trial was an online, randomised trial. Members of the public were invited to take part through social media campaigns. People were asked to either read a book in bed before going to sleep (intervention group) or not read a book in bed before going to sleep (control group). We asked everyone to do this for 7 days, after which they measured their sleep quality.

**Results:**

During December 2019, a total of 991 people took part in The Reading Trial, half (496 (50%)) in the intervention group and half (495 (50%)) in the control group. Not everyone finished the trial: 127 (25.6%) people in the intervention group and 90 (18.18%) people in the control group.

Of those providing data, 156/369 (42%) people in the intervention group felt their sleep improved, compared to 112/405 (28%) of those in the control group, a difference of 14%. When we consider how certain we are of this finding, we estimate that, in The Reading Trial, sleep improved for between 8 and 22% more people in the intervention group compared to the control group.

**Conclusions:**

Reading a book in bed before going to sleep improved sleep quality, compared to not reading a book in bed.

**Trial registration:**

ClinicalTrials.gov NCT04185818. Registered on 4 December 2019.

## Background

The COVID-19 pandemic has shown how important reliable evidence is in supporting people to make decisions about their health. This evidence includes results of randomised trials, where people take part in an experiment designed to find out if an intervention—perhaps a drug, a lifestyle change or a new approach to treatment works^1^.

Mainstream media (such as newspapers and TV programmes) and social media (such as Facebook and Twitter) often have reports and discussions about clinical trials for treatments and vaccinations. Clinical trials are now a part of the public consciousness and conversation.

Still, one of the main reasons trials are discontinued, causing research waste, is because not enough people take part in trials [1, 2], and those that do take part may not stay in the trial to the end [2].

Why is this important? Sometimes a particular number of people need to take part in a trial, so that there is enough evidence to answer the question the trial is asking. A study looking at recruitment to trials (recruitment involves asking people to take part) found that during 2011, more than 48,000 people took part in trials that could not meaningfully answer the question they were designed to answer, because the trials did not recruit enough people [3]. Similarly, a recent systematic review (which looks at many different studies) of 151 publicly funded, randomised trials found that 44% of trials did not recruit enough people to meet their target sample size [4]. This means there was not enough evidence to answer the questions the trials were asking.

Although it may be possible to combine the results of smaller trials to answer a trial question, this raises questions such as—should people take part in a study that cannot answer the question it is asking? It also raises concerns about cost—is it a waste of money and time to run a study that cannot answer the question it is asking? [5]. This is why exploring ways to encourage more people to take part in clinical trials and to keep taking part right to the end of the trial would help reduce waste of resources and money [6].

We know from research carried out before that if people have some knowledge and understanding of clinical trials, this is helpful when they are invited to take part [7, 8]. When people are confused about trials and their processes, it has the opposite effect [9–11]. Distrust and fear of research and researchers stop people from becoming involved in research projects, particularly in groups such as minority ethnic and socioeconomically disadvantaged groups [9, 10, 12]. Importantly, these groups are often missing from clinical trials, or they take part in low numbers. This means the clinical trials are not finding out evidence with and for those groups.

*The People’s Trial* was an online initiative to support and develop people’s understanding of randomised trials, in a new, easy-to-do way, by involving them in the trial research process from beginning to end.

*The People’s Trial* had seven phases and followed the steps of a randomised trial: (i) coming up with ideas for questions the trial would try to answer, (ii) narrowing down and selecting these questions, (iii) figuring out how we would answer the trial question, (iv) inviting people to take part in the trial and inviting them to self-randomise themselves to reading a book in bed (the intervention) or not reading a book in bed (the control), (v) carrying out the trial requirements (reading or not reading a book in bed before sleep) and examining the information collected during the trial, (vi) deciding how the findings of the trial would be communicated, and (vii) communicating the trial’s results.

Members of the public drove all seven phases and were the ones making key design decisions. Details on the processes underpinning *The People’s Trial* are in a separate report.

The question chosen by the public for *The People’s Trial* was:

‘Does reading a book in bed make a difference to sleep in comparison to not reading a book in bed?’

This question formed the basis of The Reading Trial.

While we did not ask people who took part in *The People’s Trial* why they chose this question, recent studies suggest that problems with sleep are increasing, with one in four people reporting that they do not sleep well [13, 14].

The most common type of sleep problems reported are difficulty trying to get to sleep, and not being able to stay asleep; these problems are often referred to as insomnia [15]. Tiredness and irritability as a result of insomnia can make everyday life harder [16–18]. Having insomnia for a long time is linked to mood disorders, such as depression and anxiety, and insomnia has also been linked to medical conditions such as high blood pressure [19].

Reading in bed before sleeping is a low-cost, accessible intervention that might improve sleep quality. The Reading Trial aimed to find out if reading a book in bed before sleep makes a difference to sleep quality, compared to not reading a book in bed before sleep.

## Methods

### Trial design and setting

The Reading Trial was an online, randomised trial with two groups. It took place on a purpose-built website (www.thepeoplestrial.ie), with no face-to-face interaction between people taking part or researchers. The Reading Trial was pragmatic, in that the intervention (reading a book in bed before going to sleep) and the comparator (not reading a book in bed) happened in ‘real-life’ conditions, for example in peoples’ own homes.

### Participants—who took part?

People who took part in The Reading Trial (the participants) were 18 years of age or over. As we could not offer a translation service, people who took part in the study also needed to be able to read about the trial and report their experiences in English.

### Participants—how we asked people to take part?

We invited people to take part in *The People’s Trial* and The Reading Trial using social media campaigns on Facebook, Twitter, Instagram and YouTube. We asked people to go to *The People’s Trial* website, where an animated video described the aim of *The People’s Trial*, and the steps involved in taking part in a randomised trial. We also included a Participant Information Leaflet on the website, which people could read and download. This gave detailed information about why we were doing the trial, what people could expect to happen if they took part in the trial and some potential risks and benefits to taking part in the trial.

After reading the Participant Information Leaflet, people who wanted to take part in The Reading Trial showed their agreement (consent) using an online form. Altogether, 991 people agreed to take part in The Reading Trial.

People taking part in the study gave us some information about themselves. This included their age group, gender, email address (so we could contact them with trial-related information), whether or not they worked in healthcare or health research, and what they felt their level of understanding of randomised trials was. People also told us about how well or poorly they felt they slept (sleep quality) in the seven nights before taking part in The Reading Trial.

### How people were divided into groups for The Reading Trial

An external developer that was not part of *The People’s Trial* team created an online program that placed (or allocated) people into the intervention group (reading a book in bed), or the control group (not reading a book in bed). Who got into which group was decided randomly by the computer program, which used something called ‘permuted block randomization with random block sizes’. This method ensures participants are placed in groups at random, while also keeping a balance across groups so that at any one time there are similar numbers of people in each group, but which group someone might be allocated to cannot be predicted in advance. Researchers involved in The Reading Trial had no access to the randomisation process at any stage of the trial. In The Reading Trial, this meant that everyone had an equal chance of being in the intervention group or the control group. We call this process randomisation.

We carried out the randomisation using a 1:1 ratio. This meant that for every person placed in the intervention group (reading a book in bed), one other person was placed in the control group (not reading a book in bed). This was to make sure The Reading Trial was a fair comparison between the two groups.

In The Reading Trial, neither the researchers nor people taking part in the trial knew in advance which group a person would be put into. Because the trial relied on people doing or not doing something, it was impossible to hide, or blind, people to the group they were placed into (reading or not reading a book) in this trial. This meant that people taking part in The Reading Trial were aware of the group that they were allocated to. However, the researchers running the trial day-to-day did not know who was in each group.

### Interventions

As part of *The People’s Trial*, members of the public designed the steps of The Reading Trial. Through an online survey, they defined the characteristics of the intervention (reading a book in bed), the comparator (not reading a book in bed), and the outcome (sleep quality). They also told us how they thought the trial should measure the outcome.

#### Intervention group

People in the intervention group:

1. Read a book for 15–30 min immediately before trying to go to sleep for seven nights in a row
2. Went to bed and woke up at the same time as they usually would
3. Did not eat food or drink caffeinated drinks (such as coffee) within 1 h of going to bed
4. Slept in their bed, in their own home, for the study duration
5. Could use electronic entertainment or communication devices (e.g. mobile phones/tablets) in bed for the seven nights of The Reading Trial.

#### Control group

People in the control group did the same as those in the intervention group, except they did NOT read a book immediately before trying to go to sleep.

This meant that people in the control group:

1. Did not read a book immediately before trying to go to sleep for seven nights in a row
2. Went to bed and woke up at the same time as they usually would
3. Did not eat food or drink caffeinated drinks (such as coffee) within 1 h of going to bed
4. Slept in their bed, in their own home, for the study duration.
5. Could use electronic entertainment or communication devices (e.g. mobile phones/tablets) in bed for the seven nights of The Reading Trial.

There were no other rules. This meant that the only difference between the intervention group and the control group was reading a book in bed, or not, for the seven nights of the study.

### Outcomes

The main or primary outcome of The Reading Trial was how well people slept, in other words, their overall sleep quality. This was measured using a scale (or questionnaire) called the ‘single item sleep quality scale (SQS) [20]. It measures sleep quality using a simple format, and when compared to longer, more complex questionnaires, this simple scale produced similar results [20].

After completing The Reading Trial, people rated their overall sleep quality using this scale, which is numbered from 0 to 10, with numbers increasing in units of one, (0 = terrible, 1–3 = poor, 4–6 = fair, 7–9 = good and 10 = excellent).

The other outcomes we measured were sleep disturbance and daytime sleepiness.

We measured sleep disturbance using the PROMIS (Patient-Reported Outcomes Measurement Information System) Short-Form Sleep Disturbance Scale. This has eight items, each on a 5-point scale, with a difference of one unit between each point on the scale. It was developed by The National Institutes of Health [21, 22]. In The Reading Trial, we used the PROMIS scale to measure how often people had problems from not having enough sleep.

Separately, we measured people’s experience of ‘daytime sleepiness’ using a 10-point scale, again the points on the scale increase in units of one. Previous studies have found this simple scale to be accurate in measuring daytime sleepiness when compared to more complex scales [23].

For all outcomes, the time we were interested in was the 7 days during which a person took part in The Reading Trial.

### Sample size—how many people did we need to get reliable results?

We wanted to be sure that we had enough people (in other words, a large enough ‘sample size’) taking part in The Reading Trial to be confident that the results were reliable. To find out how big our sample size needed to be, we searched the literature (published studies and reports) to see how common sleep disorders were reported across different countries and in different groups of people (e.g. students, older adults). We also looked at how sleep quality was reported, e.g. sleep disturbance (sleep broken by wakening) or sleep latency (the amount of time it takes you to go from being fully awake to sleeping) and sleep duration (how long you sleep). One study of 2089 people estimated that 57% of people aged 18–70 years have enough sleep [24]. This gave us some information on the number of people reporting poor quality sleep in general and, based on this information, we felt that an improvement of at least 10% on an individual’s sleep quality would be considered meaningful.

We worked out how many people we would need to take part in the trial for us to have enough information to ensure a high chance (80% or more), of being able to detect a real difference in sleep quality between the two groups, and a low chance (5% or less), of seeing a difference that was not real, or seeing a difference that happened just by chance. Using this information, we estimated that we would need at least 564 people in The Reading Trial, of which 282 would be in the intervention group (reading a book in bed) and 282 in the control group (not reading a book in bed).

To try to make it less likely that people left before the trial started, people were placed in their groups (at random) immediately after giving their consent and on the same day as they started the trial. We tried to include everyone who began the trial in our calculations. If someone left the trial, we asked that person if we could still collect information important to the study even though they were no longer taking part in the trial. We recorded all information and communication with people taking part in the trial on a database that only the research team could access. We analysed the data by looking at the outcomes (results) for people in each group who completed the outcome assessments at day 7. We also used a type of data analysis called ‘intention to treat’, which analysed the information of everybody randomised in The Reading Trial based on the groups to which they were allocated, and whether they completed the trial or not.

As The Reading Trial took place over a short time (7 days) and was a low-risk study, the steering group (a group of people that provided overall supervision of the trial), decided that we did not need a Data Management Committee for this study. The ethics committee, a committee whose role is to protect people taking part in research [25], were happy with this decision.

### Analysing information

In this section, we will describe the different ways we looked at the results of The Reading Trial in order to check if reading a book at night is likely to improve quality of sleep.

We analysed the data collected on everybody who completed The Reading Trial based on which of the two groups they were randomly allocated to. Not everyone completed the outcome assessments at day 7, in fact, 217 (21.9%) people did not. This can create problems when analysing trial data, so we filled in gaps in the data using a statistical technique called *multiple imputation*. This uses the data we do have from participants to estimate what the missing data might have been, and allowed us to see how the missing data might affect the overall results.

#### Main outcome

For The Reading Trial, we asked enough people to take part so we could detect a difference in improved sleep quality if there was any. We measured sleep quality on a scale that went from 0 to 10. This allowed us to work out whether people had improved quality of sleep, no change, or a worse quality of sleep from the start to the finish of the trial. We then compared this information for people in the intervention group (reading a book in bed) with people in the comparator group (not reading a book in bed). We looked at the data to see how certain we could be of our findings. We checked the data by creating graphs and summaries that would help us identify unusual values or results that needed further checking.

Once we were happy that the data we had collected were correct, we made graphs to help us see whether the two groups were similar at the start of the trial (i.e. that randomisation worked). We also checked if our findings were not simply a result of other differences between the groups. We did this by working out the ‘typical’, or most likely value for each measurement, and then looking at how these were different from person to person and between the intervention (reading a book in bed) and the control group (not reading a book in bed). This information is not only useful to this trial but to help design future trials that also wish to compare sleep quality.

We compared the overall sleep quality between people in the intervention group and people in the control group. We used a statistical model, called a Proportional Odds Model, that takes into account that sleep quality in The Reading Trial was measured using categories (i.e. ‘terrible’ being the worst sleep quality through to ‘excellent’ being the best sleep quality) as well as the influence of:

- Sleep quality at the start of the trial
- Gender
- Age
- Knowledge of clinical trials
- Whether the person worked in healthcare or not

As part of the main analysis, we looked at whether a person’s quality of sleep improved from the start to the finish of the trial. We compared this information for people in the intervention group with people in the control group, to see if the proportion of people with improved sleep was likely to be different in general.

#### Secondary outcomes

We looked at changes in sleep disturbance and daytime sleepiness between the intervention (reading) and control (non-reading) groups. This time we used a statistical model, called a Linear Model, which took into account that sleep disturbance and daytime sleepiness are measured as a score (i.e. sleep Disturbance was measured on a 5-point scale, daytime sleepiness was measured using a single 10-point scale). Once again, we took into account the influence of people’s sleep quality at the start of the trial, gender, age, knowledge of clinical trials and whether a participant worked in healthcare or not.

To make sure our findings were not simply a result of chance, we decided, before we did any analysis of the results, what level of certainty we would need to see in order to claim that reading a book in bed is beneficial to people similar to those who took part in The Reading Trial. The value generally used in clinical trials to represent this level is 0.05, meaning that there is a 1 in 20 chance of falsely claiming that an intervention worked. This is the value we used for The Reading Trial.

## Results

**Fig. 1:**
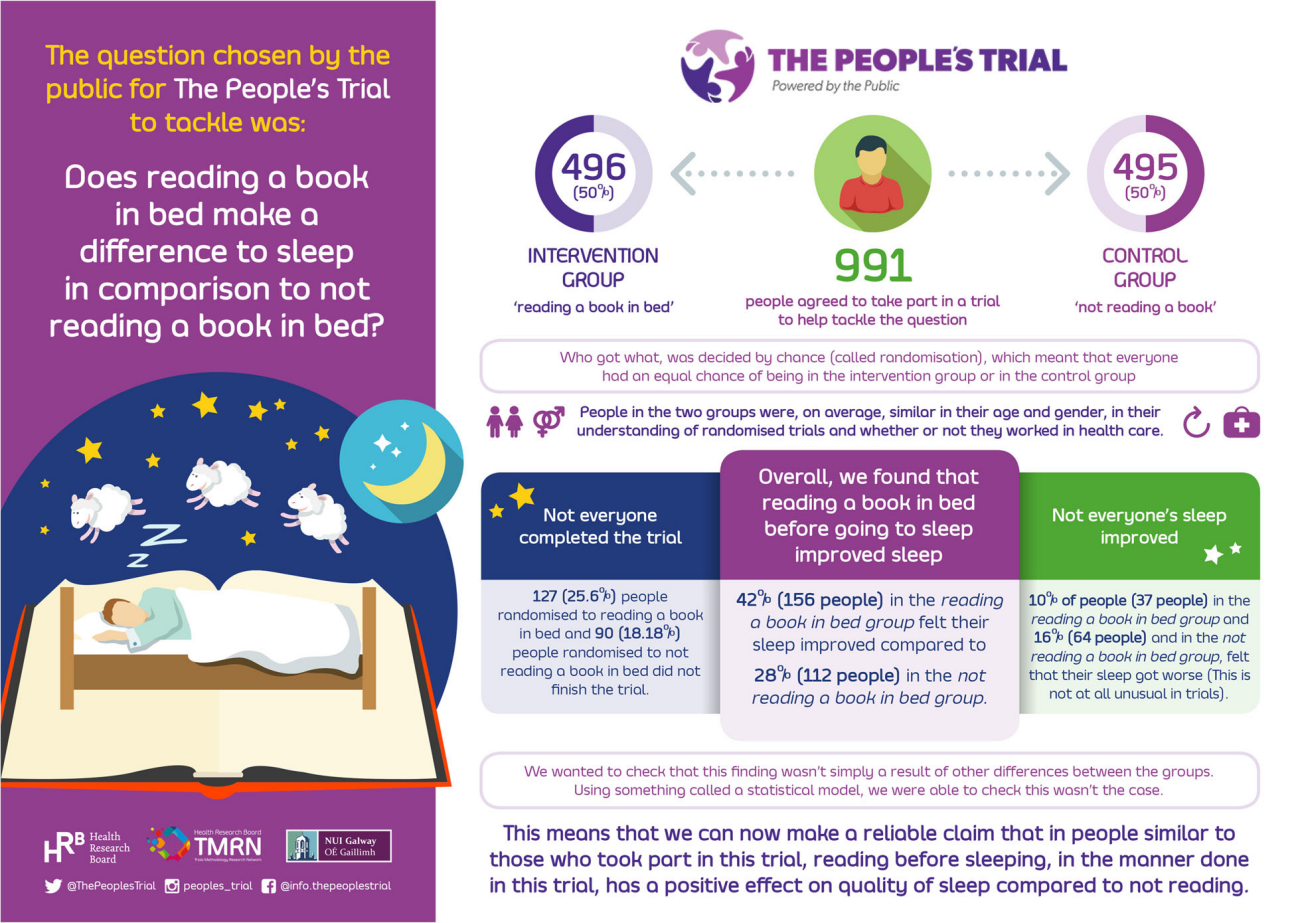
The Reading Trial results

An infographic showing the key results of The Reading Trial is shown in Fig. 1.

Between 4 December 2019 and 30 December 2019, a total of 991 people took part in The Reading Trial. These 991 people were placed into one of two groups: 496 (50%) in the ‘reading a book in bed’ group (called the intervention group) and 495 (50%) in ‘not reading a book’ group (called the control group). Although The Reading Trial needed only 564 people to reach its target sample size, *The People’s Trial* aimed to help the public learn about randomised trials, so people continued to join the trial after this number was reached.

Not everyone completed the trial. This sometimes happens in trials, even though it is something researchers would like to avoid. In this trial, 127 (25.6%) people randomised to reading a book in bed (the intervention group) and 90 (18.18%) people randomised to not reading a book in bed (the control group) did not finish the trial. This difference was large enough to provide evidence that, in general, people were more likely to drop out of the reading group rather than the control group. It is important to understand why this happened to help inform similar trials in the future. We do know that people who did not complete the trial were mainly younger (158/217 (73%) were aged 44 years or younger) or told us they did not have good sleep quality to start with (146/217 (67%) of people who did not finish the trial told us they had fair, poor or terrible sleep to begin with).

**Fig. 2:**
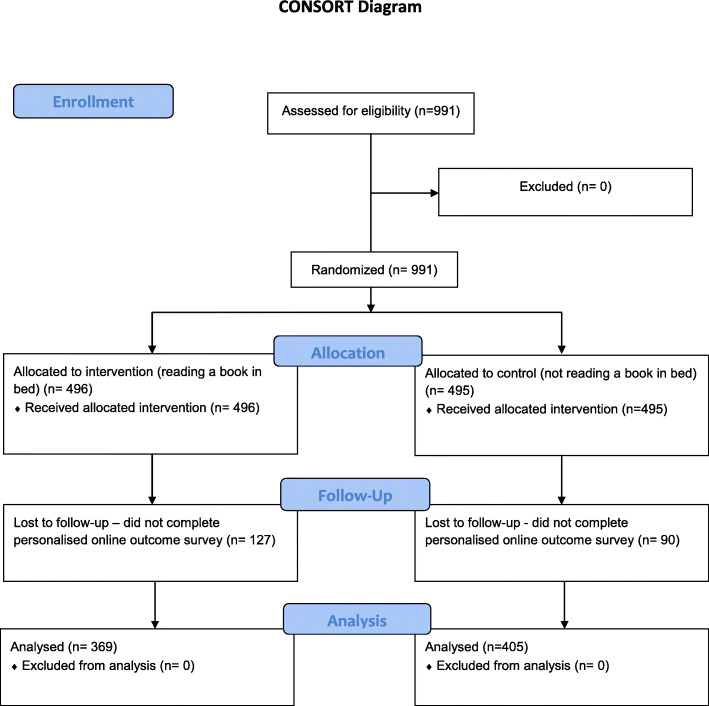
Consort flow diagram

In the end, 774 people from 43 countries; 369 (47.67%) people in the intervention group and 405 (52.33%) in the control group, stayed in The Reading Trial to the end (see Fig. 2).

The characteristics (features) of these 774 people are presented in Table 1.

**Table 1:** How similar were people in the two groups (reading a book in bed and not reading a book in bed) at the start of the trial? Data are numbers of people (%)

People who took part in The Reading Trial	Reading group (***n***=369) ***n*** (%)	Not reading group (***n***=405) ***n*** (%)
**Age**		
• 18–24 years	21 (6%)	28 (7%)
• 25–44 years	193 (52%)	209 (51%)
• 45–64 years	123 (33%)	145 (36%)
• 65 years and over	32 (9%)	23 (6%)
**Gender**		
• Female	289 (78.3%)	325 (80.2%)
• Male	75 (20.3%)	78 (19.2%)
• Prefer not to say/self-describe	5 (1.3%)	2 (0.5%)
**Understanding of randomised trials**		
• Good understanding	251 (68%)	278 (69%)
• Some understanding	101 (27%)	105 (26%)
• No understanding	17 (5%)	22 (5%)
**Healthcare background**		
• Healthcare	238 (64.5%)	269 (66%)
• Not healthcare	131 (35.5%)	136 (34%)

**Fig. 3:**
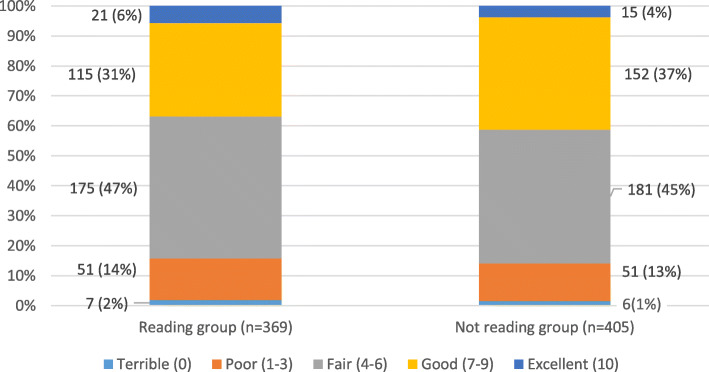
Sleep quality at the start of The Reading Trial

The characteristics of people in the two groups were, on average, similar at the start of the trial (see Table 1). Also, people in both groups told us they had similar sleep quality at the beginning of the trial (see Fig. 3). Randomisation worked to make the two groups as similar as possible at the time people joined the trial. There were some small differences. We look at what impact these might have had later in this analysis.

People rated their overall sleep quality at the start of the trial using a visual scale (from 0 to 10), which increased in units of one (0 = terrible, 1–3 = poor, 4–6 = fair, 7–9 = good and 10 = excellent).

**Fig. 4:**
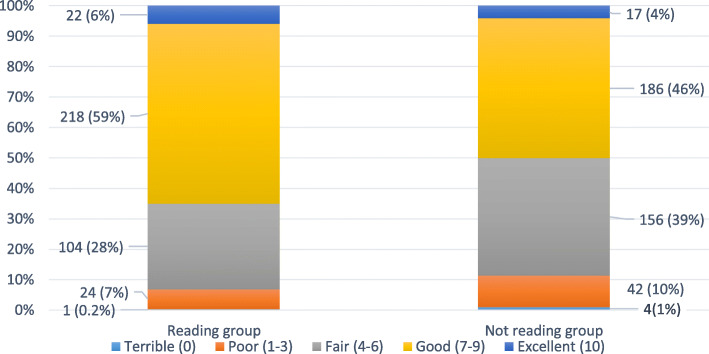
Sleep quality at the end of The Reading Trial

When we looked at the sleep quality at the end of the trial, we saw that those in the reading group tended to have better overall sleep quality (Fig. 4).

People rated their overall sleep quality at the end of the trial using a visual scale (from 0 to 10), which increased in units of one (0 = terrible, 1–3 = poor, 4–6 = fair, 7–9 = good, and 10 = excellent).

We also found that people in the intervention group (reading a book in bed) had lower sleep disturbance, on average, compared to those in the control group (not reading a book in bed) (see Table 2). However, we did find a very slight increase in the average daytime sleepiness in the reading group.

**Table 2:** What did sleep quality look like in the two groups (reading a book in bed and not reading a book in bed) at the end of the trial?

Sleep quality at the end of the trial	Reading group (***n***=369) ***n*** (%)	Not reading group (***n***=405) ***n*** (%)
• Terrible	1 (0.27%)	4 (0.99%)
• Poor	24 (6.50%)	42 (10.4%)
• Fair	104 (28.2%)	156 (38.5%)
• Good	218 (59.1%)	186 (45.9%)
• Excellent	22 (5.9%)	17 (4.25%)
Sleep disturbance^4^		
Mean^1^ (sd)^2^	46.7 (7.97)	49.9 (7.94)
Median (min, max)~	45.5 [28.9, 70.8]	50.1 [28.9, 73.0]
Daytime sleepiness^5^		
Mean (sd)	6.86 (1.93)	6.15 (2.05)
Median^3^ (min, max)	7 [ 0, 10]	7 [0, 10]

^1^Mean tells us the average sleep disturbance score indicated by people who took part in The Reading Trial

^2^The standard deviation (sd) tells us the amount of variability we found in the individual scores people reported for sleep disturbance compared to the mean score

^3^The median tell us what the ‘middle’ score was in the list of scores indicated by people when we asked them to score their daytime sleepiness after taking part in The Reading Trial

^4^We measured sleep disturbance using the PROMIS (Patient-Reported Outcomes Measurement Information System) Short Form Sleep Disturbance Scale (eight items each on a 5-point scale with a difference of one unit between each point on the scale)

^5^Daytime sleepiness’ was measured using a 10-point scale. The points on the scale increase in units of one

When we looked at each participant to see how many told us they had improved, had no change or had a worse quality of sleep from the start to the finish of the trial, we found:

- Overall, *reading a book in bed before going to sleep improved sleep quality*. In the intervention group (reading a book in bed), 42% (156 people) felt their sleep quality improved compared to 28% (112 people) in the comparator group (*not reading* a book in bed), a difference of 14% favouring the intervention group.
- When we take into account how certain we are of this finding, we estimate that the difference is likely to be between 8 and 22%.
- Although reading improved sleep quality overall, not everyone’s sleep improved. Ten per cent of people (37 people) in the reading group and 16% (64 people) in the not reading group felt that their sleep got worse.

It is highly unlikely, a probability of less than 0.001 (much less than 1 in 20 (0.05)), that we would have seen this improvement in sleep quality in people due to chance alone. This probability is called a ‘*p* value’ and is typically reported in the results of a trial. As our *p* value is less than 0.001 and therefore much less than our threshold of 0.05, we can say there was convincing evidence that people in the intervention group (reading a book in bed) were more likely to have better overall sleep quality than those in the control group (not reading a book in bed).

We did additional analysis by replacing the information that was missing for those people who were allocated to either of the two groups but who did not complete the outcome assessments when they finished the trial (21.8% (217 people)). We found that *reading a book before going to sleep still improved sleep quality.*

Not surprisingly, a person’s sleep quality at the start of the trial influenced their sleep quality at the end of the trial.

There was little evidence that a person’s gender, age, how much they knew about clinical trials or whether they worked in healthcare played an important role in sleep quality at the end of the trial.

We also found evidence that people in the intervention group (reading a book in bed) experienced less sleep disturbance compared to people in the control group (not reading a book in bed). We found that sleep disturbance is likely to be lower, on average, by between 2 and 4 units when reading a book in bed before sleeping. As sleep disturbance is recorded on a scale from 1 to 100, this is the same as saying that sleep disturbance is likely to be lower by 2% to 4% in those that read a book before sleeping.

We found that daytime sleepiness is likely to be higher, on average, by between 0.5 and 1 unit for people in the intervention group (reading a book in bed). As daytime sleepiness is recorded on a scale from 1 to 10, this is the same as saying that daytime sleepiness is likely to be higher by 5% to 10% in those that read a book before sleeping.

Even though sleep disturbance was lower in the intervention group (reading a book in bed) compared to the control group (not reading the book), the difference is small and likely to have little impact practically. Similarly, although daytime sleepiness was higher in the intervention (reading) group, again the increase was very small and likely to have little impact.

### Serious adverse events

The question The Reading Trial explored included a familiar, accessible, low-risk intervention (reading a book in bed) and comparator (not reading a book in bed). Using an 'everyday’ intervention lessened the risk of people experiencing any harm from the intervention. Using a common intervention also made the question relevant for a wider group of people. No adverse (negative or harmful) events were reported by people taking part, either during The Reading Trial or in the follow-up period.

## Discussion

Sleep problems are relatively common, with one in four people reporting that they do not sleep well. This causes different issues for different people: for some, it is difficulty getting to sleep and for others, it is staying asleep. However, despite being a common problem that can severely affect a person’s quality of life, it is often under-diagnosed and under-reported to health care providers [26, 27]. When it is reported, the most common treatment for insomnia (difficulty in getting to sleep or staying asleep for long enough) is medicine [15]. It is estimated that approximately one-third of adults age 50 and older in the USA take sleep medication [28]. While non-drug sleeping aids are available, such as cogitative behaviour therapy (CBT), these often require significant time and commitment [28]. They can also be costly.

The Reading Trial looked at a low-cost, accessible intervention that might affect sleep quality. The Reading Trial showed that in a group of people similar to those who took part in the trial, reading a book in bed before sleeping improves sleep quality, compared to not reading a book in bed. We found that reading in bed before sleep not only potentially improves overall sleep quality but also people in the reading group experienced fewer problems staying asleep. While we did find a higher rate of daytime sleepiness in people allocated to reading a book in bed, the difference we found was very small and likely to have little impact on a person’s daytime sleepiness in practice.

Recent studies highlight the positive effects of public and patient involvement in clinical trials. The benefits include increased health literacy and knowledge of trial processes [29, 30]. People who took part in The Reading Trial experienced the process of randomisation themselves and discovered through a lived experience why this is important. They learnt what makes a ‘good’ trial question and thought about how we might carry out the trial, how we could identify and measure outcomes and how we might best share the trial results.

The Reading Trial had a number of strengths. Many trials measuring the effect of an intervention on sleep quality do so in specific groups of people, e.g. cancer patients [31], older adults [32] or those with a history of mental health diagnosis [33]. The Reading Trial included a large, diverse sample of participants. This helped the trial to provide a more accurate measure of the effect of reading a book in bed on overall sleep quality in the general public.

We used a randomised trial design, which is considered the best way to measure the effect of an intervention. The public showed, through an online survey, how they wanted us to measure the outcome ‘sleep’. We prioritised the outcomes (overall sleep quality, sleep disturbance and daytime sleepiness) based on this response, leading us to choose ‘overall quality of sleep’ as the primary outcome, the most important thing we wanted to measure in the trial. We also used an online format to invite people to take part in the trial and we ran the trial online, which meant that lots of people could take part in clinical research.

As with any study, The Reading Trial had some limitations. We measured the effect of reading a book in bed on sleep quality for seven nights. We do not know if continuing to read in bed before sleep for longer than seven nights would increase, decrease, or maintain the effect we found on overall sleep quality in the trial. Not everyone completed the trial, with people in the reading group more likely to not complete the trial than those in the control group. We do not know the reasons why some people did not finish The Reading Trial and this information would support further research in this space.

Another limit is that people who took part in The Reading Trial told us the effect, if any, the intervention (reading a book in bed) made to their sleep using an online questionnaire. When people self-report the effects of an intervention in this way, they may over or under-estimate the true effect of the intervention. This is known as response bias, and it occurs when the person completing a questionnaire, mistakenly tries to make themselves or the intervention appear ‘better’, even when the survey is anonymous [34, 35].

While online trials are becoming more common, we believe that The Reading Trial was special. It was a trial designed *by the people, for the people*. The Reading Trial needed people to get involved and create each step of the trial process. It would be successful only if people embraced the trial they had created. While *The People’s Trial* offered people an opportunity to learn about randomised trials, its little sister, The Reading Trial, offered almost one-thousand people the experience of actually taking part in a trial, in a low risk, accessible environment.

Moreover, we believe the results. The Reading Trial was a real trial following standards those who work professionally in trials would recognise. Involving the public directly in design decisions does not compromise rigour, but it does increase relevance.

## Conclusion

Overall, we found that reading a book in bed before sleeping, in the manner done in this trial, improves the quality of sleep compared to not reading a book in bed before sleeping. Getting people to take part in randomised trials can be difficult. Supporting public knowledge—and public understanding of the reasons why we do randomised trials and why they are important—has a positive impact on public engagement. Involving the public directly in design decisions as done in The People’s Trial, helps not only public understanding but improves our trials.

## Data Availability

All data and materials are available from the corresponding author on reasonable request.

## Footnotes

1 Randomised trials are a type of research study that compares groups of people receiving different interventions and looks at which of these improves health outcomes the most. An intervention is anything that aims to make a change to someone’s health such as drugs, surgical procedures, or lifestyle changes. In a randomised trial the decision about which group a person joins in a trial is made randomly, which means that people have an equal chance of being placed into any group.
